# Virus diversity and interactions with hosts in deep-sea hydrothermal vents

**DOI:** 10.1186/s40168-022-01441-6

**Published:** 2022-12-24

**Authors:** Ruolin Cheng, Xiaofeng Li, Lijing Jiang, Linfeng Gong, Claire Geslin, Zongze Shao

**Affiliations:** 1 Key Laboratory of Marine Genetic Resources, Third Institute of Oceanography, Ministry of Natural Resources, Xiamen, China; 2 State Key Laboratory Breeding Base of Marine Genetic Resource, Fujian Key Laboratory of Marine Genetic Resources, Xiamen, 361005 China; 3 State Key Laboratory for Managing Biotic and Chemical Threats to the Quality and Safety of Agro-Products, Key Laboratory of Biotechnology in Plant Protection of Ministry of Agriculture and Zhejiang Province, Institute of Plant Virology, Ningbo University, Ningbo, 315211 China; 4 Univ Brest, CNRS, IFREMER, IRP 1211 MicrobSea, Laboratoire de Microbiologie des Environnements Extrêmes LM2E, IUEM, Rue Dumont d’Urville, F-29280 Plouzané, France; 5 Sino-French Laboratory of Deep-Sea Microbiology (MICROBSEA-LIA), Plouzané, France; 6 Southern Marine Science and Engineering Guangdong Laboratory (Zhuhai), Zhuhai, 519000 China

**Keywords:** Deep-sea hydrothermal vent, Viruses, Metagenomes, Viral ecology

## Abstract

**Background:**

The deep sea harbors many viruses, yet their diversity and interactions with hosts in hydrothermal ecosystems are largely unknown. Here, we analyzed the viral composition, distribution, host preference, and metabolic potential in different habitats of global hydrothermal vents, including vent plumes, background seawater, diffuse fluids, and sediments.

**Results:**

From 34 samples collected at eight vent sites, a total of 4662 viral populations (vOTUs) were recovered from the metagenome assemblies, encompassing diverse phylogenetic groups and defining many novel lineages. Apart from the abundant unclassified viruses, tailed phages are most predominant across the global hydrothermal vents, while single-stranded DNA viruses, including Microviridae and small eukaryotic viruses, also constitute a significant part of the viromes. As revealed by protein-sharing network analysis, hydrothermal vent viruses formed many novel genus-level viral clusters and are highly endemic to specific vent sites and habitat types. Only 11% of the vOTUs can be linked to hosts, which are the key microbial taxa of hydrothermal habitats, such as *Gammaproteobacteria* and *Campylobacterota*. Intriguingly, vent viromes share some common metabolic features in that they encode auxiliary genes that are extensively involved in the metabolism of carbohydrates, amino acids, cofactors, and vitamins. Specifically, in plume viruses, various auxiliary genes related to methane, nitrogen, and sulfur metabolism were observed, indicating their contribution to host energy conservation. Moreover, the prevalence of sulfur-relay pathway genes indicated the significant role of vent viruses in stabilizing the tRNA structure, which promotes host adaptation to steep environmental gradients.

**Conclusions:**

The deep-sea hydrothermal systems hold untapped viral diversity with novelty. They may affect both vent prokaryotic and eukaryotic communities and modulate host metabolism related to vent adaptability. More explorations are needed to depict global vent virus diversity and its roles in this unique ecosystem.

Video Abstract

**Supplementary Information:**

The online version contains supplementary material available at 10.1186/s40168-022-01441-6.

## Background

Deep-sea hydrothermal vents are one of the most extreme and dynamic environments on Earth [1]. In this dark world, mixing between anoxic hydrothermal fluids and oxic cold seawater results in wide chemical and thermal gradients, providing energy sources for the vent ecosystems [2]. Unlike most ecosystems that are fueled by photosynthesis, biological productivity is primarily driven by chemoautotrophs in deep-sea hydrothermal vents [3]. Chemoautotrophs use the energy produced by the oxidation of sulfur, hydrogen, methane, ammonia, or iron to fix carbon [2, 4], converting dissolved inorganic carbon into the organic phase within the biota. Diffuse vent fluids are hot spots of primary productivity in the deep ocean and provide a window into the subseafloor microbial habitat [5, 6]. Hydrothermal fluids are highly diluted in plumes, which can rise hundreds of meters and disperse hundreds of kilometers away from their source and impact broader deep-sea microbial communities and biogeochemistry [7]. Over the past decade, significant efforts have been made to explore the source, diversity, and function of the microbes inhabiting hydrothermal vents [3, 5–12]. These studies have suggested that the prokaryotic communities in hydrothermal plumes are distinct from those in diffuse fluids [13] and hydrothermal sediments [7, 10]. As important components of hydrothermal vent microbiomes, viral communities have received less attention.

Viruses are the most abundant, pervasive, and genetically diverse biological entities in the biosphere [14]. In the ocean, the total estimated number of viruses is approximately 10^30^, making up the second largest relative biomass (but the most abundant) in comparison with prokaryotes and protists, despite their small size [15, 16]. They play a pivotal role in marine ecosystems not only by lysing their hosts but also through horizontal gene transfer and manipulating host metabolism via the expression of viral-encoded auxiliary metabolic genes (AMGs) [17]. Each day, viruses in surface waters kill 20–40% of prokaryotes and release up to 10^9^ tons of carbon and other nutrients, which has a significant influence on ocean biogeochemical cycles [16]. Additionally, it is estimated that marine viruses transduce approximately 10^14^–10^17^ Gbp of DNA per day [18], affecting host diversity and function. Comparatively, our knowledge of viral diversity and processes in the deep sea is quite limited, partially due to the difficulties in obtaining and processing samples from the deep sea.

In deep-sea hydrothermal vent ecosystems, virus-like particles are more abundant than prokaryotes and are believed to have a profound impact on microbial communities [19]. The viral abundances of hydrothermal plume samples were reported to be 10^5^–10^6^ VLPs ml^−1^, higher than in the surrounding seawater samples [20, 21]. Moreover, it has been suggested that hydrothermal vent microbes harbor substantial populations of temperate viruses [22–24], which may improve host fitness and facilitate horizontal gene transfer. A well-known example of phage AMGs is the gene encoding the reverse dissimilatory sulfite reductase (*rdsr*) [25]. The alpha (*rdsrA*) and gamma (*rdsrC*) subunit genes of this enzyme were identified in hydrothermal plume phages that putatively infect sulfur-oxidizing bacteria, suggesting that viruses play a direct role in the sulfur cycle. The AMGs of hydrothermal vent viruses are also involved in many other metabolic pathways, including nitrogen, methane metabolism, and amino acid biosynthesis [24, 26], or they may even compensate for novel metabolic pathways for their host microorganisms [19]. These findings suggest that hydrothermal vent viruses are a large reservoir of genetic diversity and have complex interactions with their hosts and habitats, which remain to be fully elucidated.

To date, microbes identified in hydrothermal vent habitats have largely remained uncultured, and few virus isolates have been reported [27, 28]. Instead, metagenomics technology is commonly applied to characterize the diversity, ecology, and evolution of hydrothermal vent viruses [19, 22–25, 29–31]. Some of these studies have tried to identify viruses in the cellular fractions (0.22 μm filtered), especially in recent years [29–31], because many bioinformatic tools now enable us to recover and analyze viral sequences from complex metagenomes that are generated without viral particle enrichment [32]. The cellular metagenomes may contain sequences of integrated/extrachromosomal proviruses, viruses undergoing the lytic cycle, large virions that are retained on filters, and small virions that are adsorbed onto filters, providing new insights into viral communities and virus-host interactions [29, 33–35].

In this study, we sought to obtain a comprehensive view of viral populations and their interactions with hosts in deep-sea hydrothermal vents. For this purpose, metagenomic data were generated from plume and background seawater samples collected at two hydrothermal fields on the Carlsberg Ridge (CR), northwest Indian Ocean. In addition, 29 publicly available metagenomes from different hydrothermal vent habitats around the world were compiled for analysis. Here, we provide a characterization of the community structure, the virus-host associations, and the potential ecological roles of viruses in deep-sea hydrothermal vents across the global oceans. A collection of hydrothermal vent viral genomes was recovered from microbial metagenomic datasets, and the unique features of the vent viral communities were revealed. To date, this is the largest survey of viruses inhabiting deep-sea hydrothermal vents, and the results will expand our understanding of viral diversity and functions in extreme marine ecosystems.

## Results and discussion

### Overview of deep-sea hydrothermal vent microbial communities

**Fig. 1:**
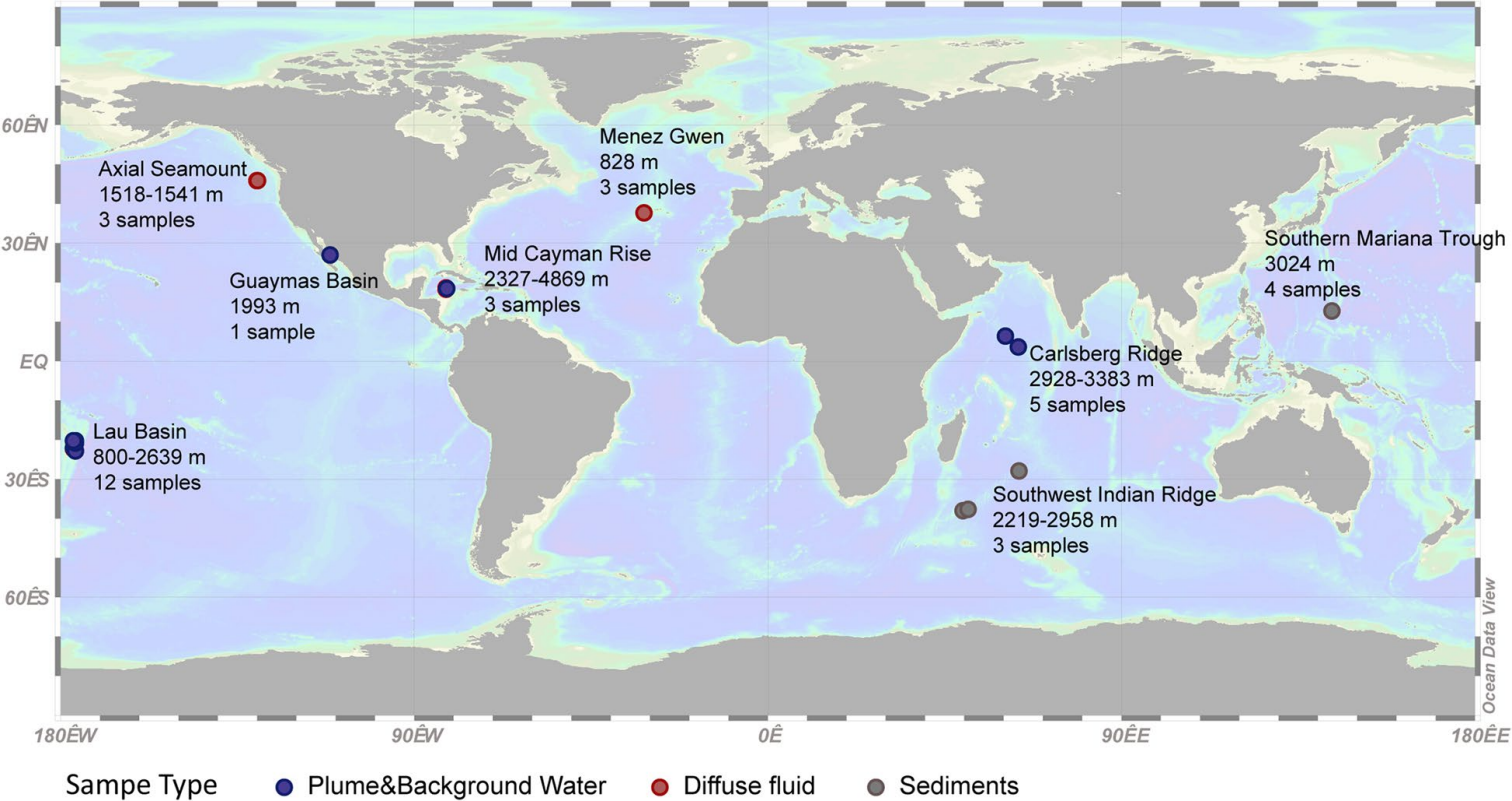
Geographic distribution of metagenome sampling sites involved in this study. The map of the sampling locations was created in Ocean Data View v5.5.2 (https://odv.awi.de/). The site names, sample type, and sampling depth range in meters below sea level (m) are shown

To explore the community composition of microorganisms in the CR hydrothermal vents, metagenomes were sequenced from five plume and background seawater samples collected at the “Wocan” and “Tianxiu” hydrothermal fields (Supplementary Table 1). For comparison, metagenomes of 29 other samples derived from seven different hydrothermal vent sites in the Pacific Ocean, Atlantic Ocean, and southwest Indian Ocean were retrieved from the public database (Supplementary Table 1). These sites are abbreviated as follows: Axial Seamount (Axial), Southwest Indian Ridge (SWIR), Menez Gwen (Menez), Eastern Lau Spreading Center (Lau), Guaymas Basin (Guaymas), Mid-Cayman Rise (Cayman), and Southern Mariana Trough (Mariana). The final datasets include 34 metagenomes representing four different types of habitats in hydrothermal environments, i.e., vent plumes (*n* = 16), background seawater (*n* = 5), diffuse fluids (*n* = 6), and sediments (*n* = 7). The sampling locations are shown in Fig. 1.

The 16S rRNA gene fragments (16S miTags) were extracted from clean reads of these metagenomes for taxonomic profiling [36]. Classification of the 16S miTags at the phylum level (the class level for *Proteobacteria*) revealed dominance by *Gammaproteobacteria* in almost all of the metagenomes, followed by *Deltaproteobacteria*, *Alphaproteobacteria*, and *Bacteroidetes* (Supplementary Fig. 1A). *Campylobacterota* (previously named Epsilonproteobacteria [37, 38]) showed high relative abundances in diffuse fluid and some plume metagenomes. The dominant archaeal lineage was *Thaumarchaeota*, which occurred primarily in seawater and hydrothermal plumes. Among the samples, taxonomic composition at the phylum level was more similar between samples from the same hydrothermal vent site, and samples from the same habitat type tended to be clustered together. It should be noted that the plume samples from CR (this study) and Lau [11] were processed by multiple displacement amplification (MDA). To assess the bias introduced by the MDA process, we compared the taxonomic composition inferred from CR metagenomic data to those from 16S rRNA gene pyrosequencing (Jiang et al., unpublished). Similar to previous observations in Lau plume samples [11], no significant differences were found in the abundance patterns, suggesting that MDA bias did not obscure the major trends.

De novo assembly and binning of these metagenome sequencing data resulted in the reconstruction of 581 high- or medium-quality prokaryotic metagenome-assembled genomes (MAGs) [39] with ≥ 50% completeness and ≤ 10% contamination. These MAGs were clustered at 95% average nucleotide identity (ANI), representing species-level groups spanning 54 phyla, including 515 bacterial and 66 archaeal MAGs (Supplementary Fig. 1B). Most of the bacterial MAGs belong to dominant lineages, such as *Gammaproteobacteria* (*n* = 138), *Bacteroidetes* (*n* = 54), *Deltaproteobacteria* (*n* = 53), and *Campylobacterota* (*n* = 41), while archaeal MAGs are primarily affiliated with *Candidatus* Thermoplasmatota (*n* = 25) and *Thaumarchaeota* (*n* = 18). These MAGs, in addition to 440 high-quality single-cell amplified genomes (SAGs) retrieved from the CR hydrothermal vents and the publicly available genomes of hydrothermal vent microbial isolates, provide a good basis for investigating the connections between viruses and prokaryotes.

### Diversity and phylogeny of hydrothermal vent viruses

VirSorter v1.0.6 [40] and VIBRANT v1.2.0 [41] were used to identify viral contigs in the hydrothermal vent metagenome assemblies, followed by manual curation. Contigs ≥ 5 kb or ≥ 2 kb and circular were pooled together, resulting in 8847 putative viral sequences. We retained the small circular contigs because these may represent small single-stranded DNA (ssDNA) virus genomes (such as phages in the family Microviridae and eukaryotic viruses in the families Circoviridae, *Geminiviridae*, and Smacoviridae). The 8847 candidate viral contigs were then clustered at 95% ANI over 80% of the sequence length, producing 4662 species-level viral populations (viral operational taxonomic units (vOTUs)) [10] (Supplementary Table 2). These vOTUs ranged from 2000 to 226,341 bp in size (total length of the vOTU contigs = 37,751,900 bp and N50 of the vOTU contigs = 10,916 bp). The largest contig has a length of more than 200 kb and might be classified as a “huge phage” [42] rather than a nucleocytoplasmic large DNA virus (NCLDV) [43], according to the presence of phage-specific genes [42]. As determined by CheckV [44], almost half of the vOTUs (47%) represented viral genomes of medium quality and above (Supplementary Fig. 2A). Based on the presence of terminal repeats and provirus integration sites, 1731 vOTUs were identified as complete viral genomes (37%). Of these, 1727 vOTUs were circular and were probably free viruses, while 4 vOTUs were predicted to be intact proviruses.

**Fig. 2:**
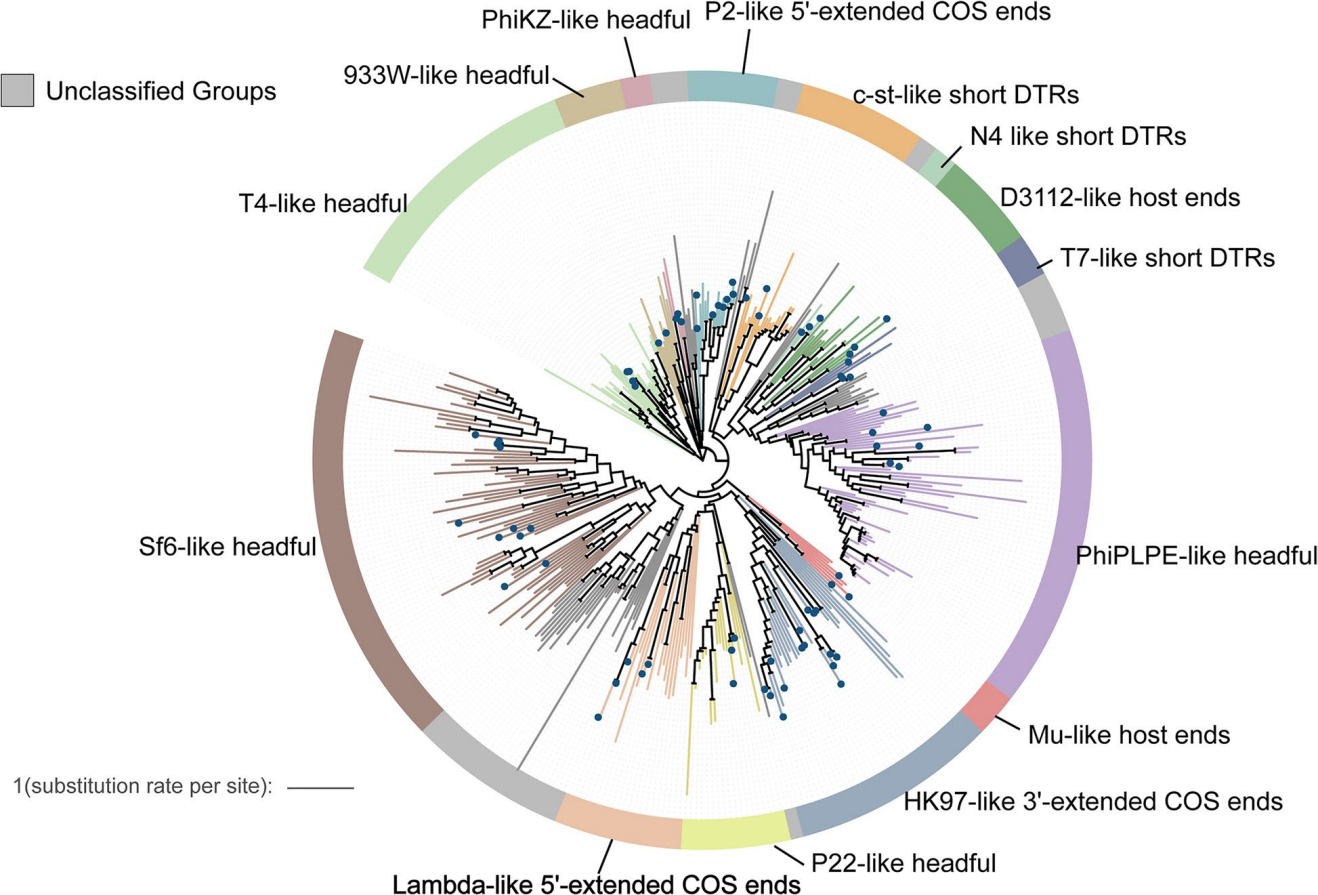
Phylogeny of the head-tail phages in hydrothermal vents. A maximum-likelihood tree was constructed using an alignment of the terminase large subunit protein (TerL) sequences of *Caudovirales*. Representative members of previously reported virus groups are indicated as blue dots, and unclassified clades are colored gray. The outer ring represents the phage DNA packaging strategies by color

A large proportion of the hydrothermal vent vOTUs were classified as double-stranded DNA (dsDNA) viruses of the order *Caudovirales* (45%), of which the family *Myoviridae* was predominant (Supplementary Fig. 2B). Because the terminase large subunit (TerL) gene is conserved in all head-tail phages [45], a phylogenetic analysis of the TerL gene was performed to assess the diversity and genetic distance of *Caudovirales* in hydrothermal vents. Within the viral contigs, a total of 638 complete ORFs encoding the TerL genes were identified and used to construct the phylogenetic tree (Fig. 2). Most of the sequences from hydrothermal vent metagenomes fell into 14 known lineages, which were defined by different DNA packaging strategies, while the rest of them formed several novel branches, indicating the remarkable diversity of head-tail phages in our datasets.

**Fig. 3:**
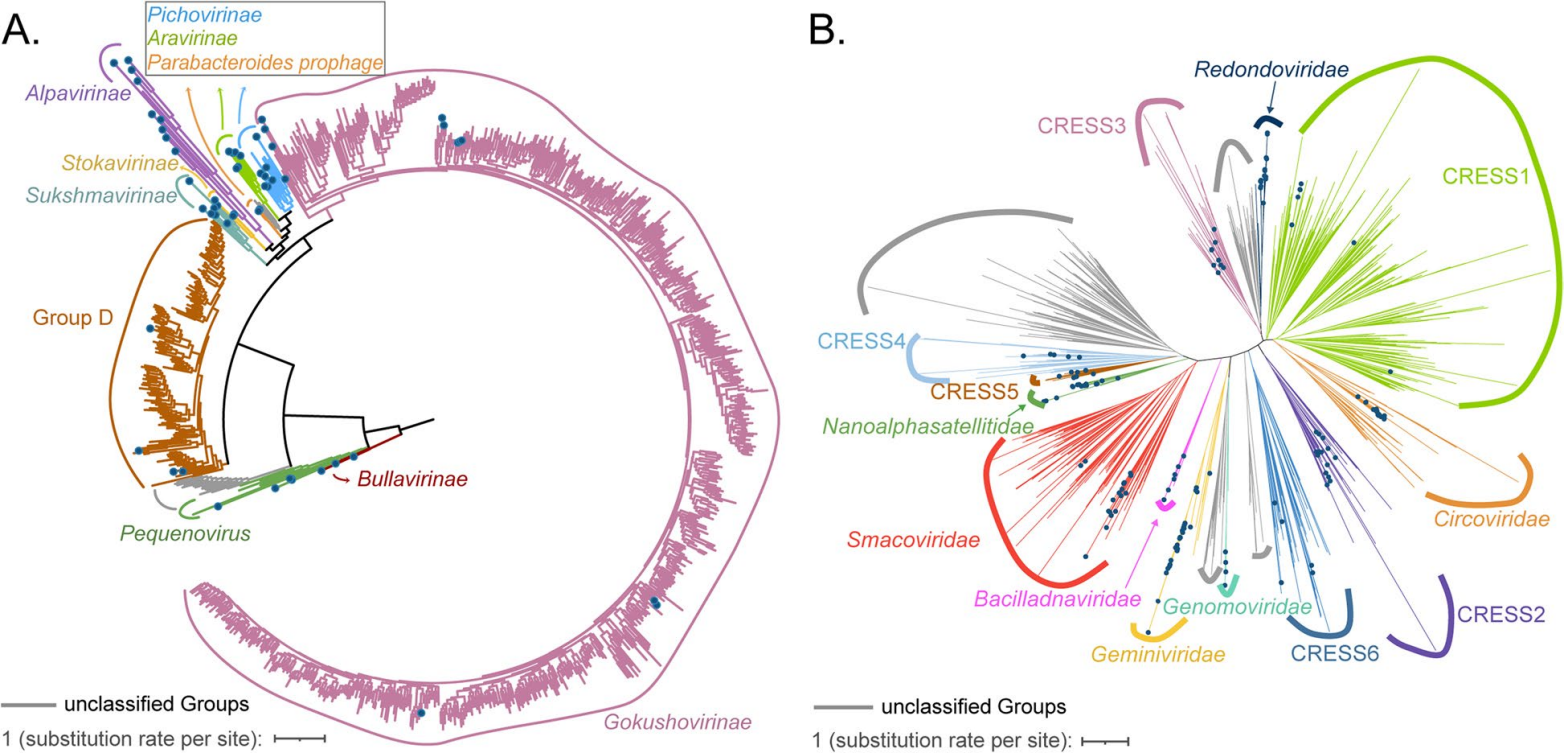
Phylogeny of ssDNA viruses in hydrothermal vents. Representative members of previously reported virus groups were indicated as blue dots, and unclassified clades were colored gray. **A** Maximum-likelihood tree based on the VP1 amino acid sequence of Microviridae. **C** Maximum-likelihood tree based on Rep proteins of CRESS-DNA viruses

ssDNA viruses accounted for approximately 36% of the total set of vOTUs, with primary assignment to the family Microviridae. Phages in the family Microviridae are among the smallest DNA viruses [46], with circular genomes ranging from 4.2 to 6.5 kb [47, 48]. A total of 872 complete or near-complete genomes of Microviridae were recovered from hydrothermal vent metagenomes. The amino acid sequences of the well-conserved major capsid protein VP1 were used as a phylogenetic marker for the classification of these viruses. The majority of the VP1 sequences showed < 70% shared identities relative to their best matches in NCBI’s NR database and showed < 70% shared identities compared to each other, reflecting high levels of divergence. The phylogenetic analysis showed that most (694 genomes) of the hydrothermal vent-derived Microviridae belonged to the subfamily Gokushovirinae, followed by group D (143 genomes). A few sequences were clustered within the clades *Pequenovirus* (8 genomes), *Pichovirinae* (4 genomes), Bullavirinae (1 genome), and Alpavirinae (1 genome), and the other 22 genomes were not clustered with any known subfamilies (Fig. 3A). Interestingly, the new clade seemed to consist of viruses with small genomes (approximately or less than 4 kb). One of these has a genome size of 3559 bp and encodes only three putative ORFs, including a capsid protein, a replication initiator, and a protein of unknown function. It represents the smallest microvirus recovered in our datasets and the smallest ssDNA phage with the least ORFs reported to date.

CRESS-DNA (circular rep. encoding single-stranded DNA) viruses, including Circoviridae and its related families, were also highly represented (15% of the vOTUs, Supplementary Fig. 2B, Supplementary Table 2). This group of eukaryotic viruses has small circular genomes and commonly encodes only 2 proteins, of which the replication initiation protein (Rep) is the only universally conserved gene [49]. Based on the sequences of Rep proteins, 694 CRESS-DNA viruses identified from hydrothermal vent metagenomes were clustered within 13 known families, while the other 145 sequences defined several potentially new clades (Fig. 3B).

The NCLDVs are another group of eukaryotic viruses [43], including the families *Poxviridae*, *Iridoviridae*, Ascoviridae, Asfarviridae, Marseilleviridae, Mimiviridae, and Phycodnaviridae, as well as several lineages of unclassified viruses. Viruses in this group were also present in our hydrothermal vent metagenomics datasets (Supplementary Fig. 2B, Supplementary Table 2). As shown in Supplementary Table 2, 27 vOTUs were classified as viruses from the Mimiviridae, Phycodnaviridae, or other NCLDV families. However, the sizes of these vOTUs ranged from 6112 to 24,611 bp and only represented small genome fragments of the viruses. Approximately 19% of the total vOTUs did not show any significant sequence similarity to any known viral families and could not be taxonomically classified for the time (Supplementary Fig. 2B).

It should be noted that all the hydrothermal plume, diffuse fluid, and background seawater samples used in this study were passed through 0.22 μm filters (Supplementary Table 1 and references therein). Giant viruses, integrated proviruses, and actively infecting viruses within the cells would be retained on the membrane, while most free virus particles of small size would be lost during this step, such as the non-tailed ssDNA viruses. Thus, we analyzed the metagenomes of the cellular fraction and the virus-like particle (VLP) fraction of the sediment samples from the southwest India Ridge [19] to evaluate the extent to which they reflect viral diversity. The results showed that the cellular fraction was comparable to the VLP fraction with respect to viral recovery (data not shown). The number of recovered CRESS-DNA viruses in VLP fractions doubled those recovered in cellular fractions, but the number of microviruses recovered in cellular fractions was greater than those recovered in VLP fractions, despite their small size. One explanation for these differences is that the intracellular microviruses were captured on filters, while most of the CRESS-DNA viruses replicating in eukaryotic hosts were excluded from sampling. No significant difference was observed for other viral groups. For hydrothermal plume and diffuse fluid samples involved in this study, metagenomic data on the viral fractions were not available. However, a comparative analysis of the cellular and viral metagenomes derived from a fluid sample was performed in a previous study [24], and high enrichment of mobile elements and proviruses was observed in the cellular fraction. Given that a high proportion of viruses in hydrothermal ecosystems are lysogenic [22–24], we supposed that the viral sequences identified in the cellular metagenomes could represent the diversity of hydrothermal viruses to a large extent. However, to fully characterize the viromes in deep-sea hydrothermal vents, the metagenomes of both the VLP fraction and cellular fraction are still needed, preferably with the addition of RNA-seq data, which will enable us to discover and analyze RNA viruses.

### Viral communities across different zones of hydrothermal vents

**Fig. 4:**
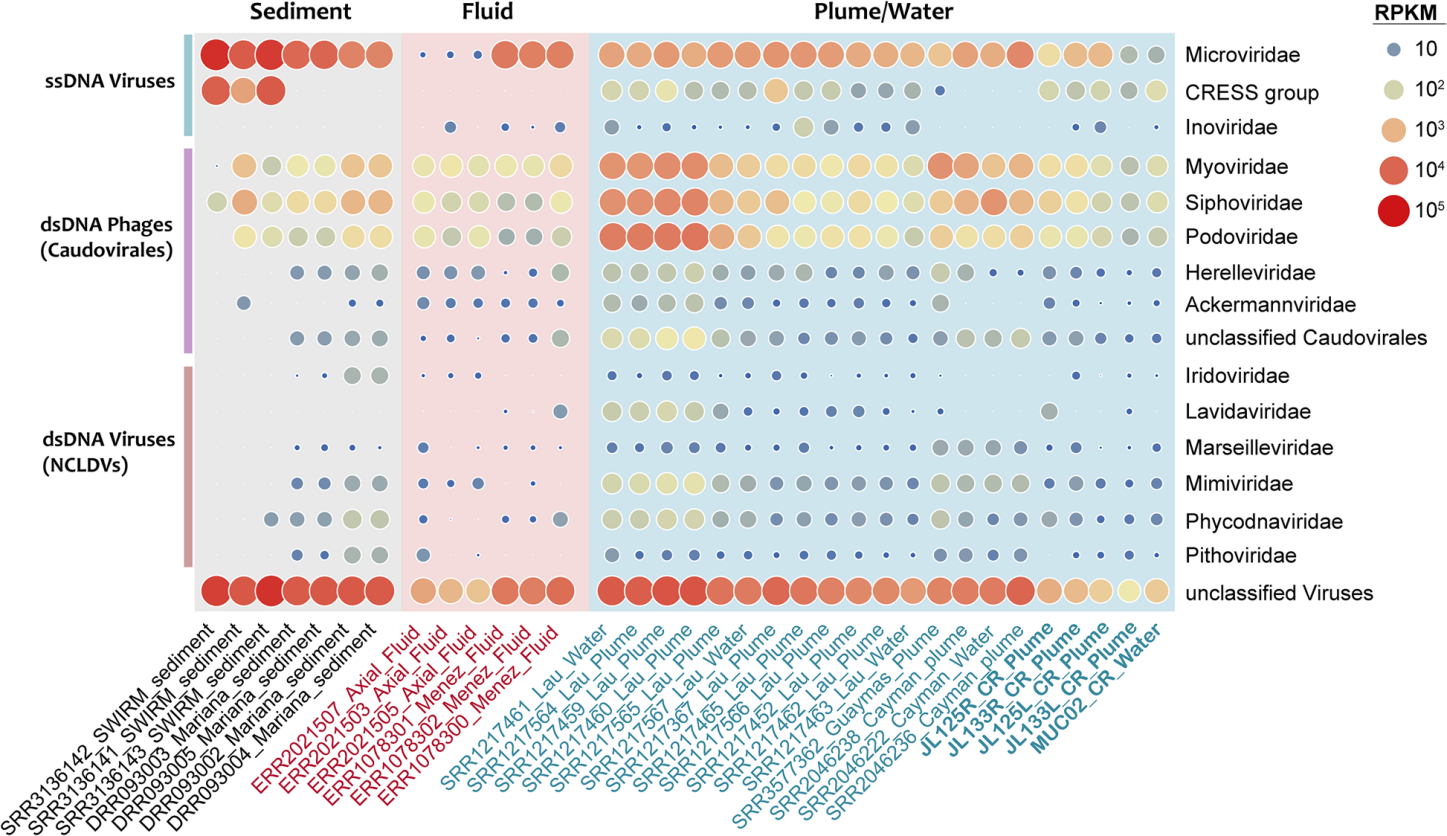
Bubble plot showing the relative abundance of viral groups in different hydrothermal samples. The metagenomic samples from this study are indicated in bold

To investigate the viral community structure in hydrothermal ecosystems, the relative abundances of vOTUs in each metagenome were calculated and normalized (Fig. 4, Supplementary Fig. 3). The 34 samples used in this study were collected from 8 different hydrothermal vents across various geographical zones, including hydrothermal plume, background water, diffuse fluid, and sediment samples. As a result, the vOTU abundance patterns were primarily clustered by sample types and secondarily by hydrothermal vent sites (Supplementary Fig. 3). The viral community composition of hydrothermal vent sediments was significantly different from that of other habitats. Hydrothermal plumes and the surrounding deep-sea water samples showed similar vOTU abundance patterns, as plume and water samples from the same hydrothermal vent field were always clustered together. This result is consistent with previous studies suggesting that plume microbial communities resemble those from background seawater samples [7], indicating that hydrothermal plumes are strongly influenced by ambient seawater. Overall, these results showed that the virome structures varied across different hydrothermal vent habitats and different hydrothermal vent fields. The hierarchical clustering of samples based on vOTU and 16S miTags showed similar patterns (Supplementary Fig. 1A, Supplementary Fig. 3), implying a close link between viral and prokaryotic communities.

Similar to other studies of marine viromes, the majority of the hydrothermal vent viral communities were composed of unclassified viruses (up to 69% of the total viral reads, Fig. 4). At the family level, the tailed phage *Myoviridae* (on average 9.2%, 7.8%, and 1.5% of plume, fluid, and sediment samples, respectively) was the most dominant group of dsDNA viruses in most samples, followed by Siphoviridae (9.1%, 4.6%, and 2.4%) and Podoviridae (7.6%, 4.8%, and 0.7%).

The ssDNA viruses also accounted for a large fraction of viral communities. In all of the sediment metagenomes, the relative abundance of Microviridae was higher than that of any other dsDNA virus family. The CRESS-DNA virus group was present in most plume samples and three of the sediment samples, accounting for approximately 2.2% of the total viral reads (Fig. 4). It is possible that the enrichment of ssDNA viruses in these datasets was caused by the MDA process, which used phi29 DNA polymerase and preferentially amplified small circular ssDNA molecules [50, 51]. However, the quantification of viral DNA without amplification also revealed the dominance of ssDNA viruses in the total DNA viral assemblages of deep-sea sediments [52]. Thus, we suggest that ssDNA viruses are abundant and play an important role in hydrothermal vent environments.

The eukaryotic NCLDVs accounted for 0.4% of the total viral reads in hydrothermal plume samples, on average. Of these families, Mimiviridae and Phycodnaviridae are the most abundant. In contrast, samples from diffuse fluid and sediment contained fewer NCLDVs. Large DNA viruses in this group have been frequently detected in marine metagenomes, including those derived from hydrothermal vents [30, 53–55]. The NCLDV sequences in metagenomic datasets may come from marine unicellular eukaryotes or free giant viruses, but their roles in these ecosystems are largely unknown.

### Hydrothermal vent viruses are novel and endemic

**Fig. 5:**
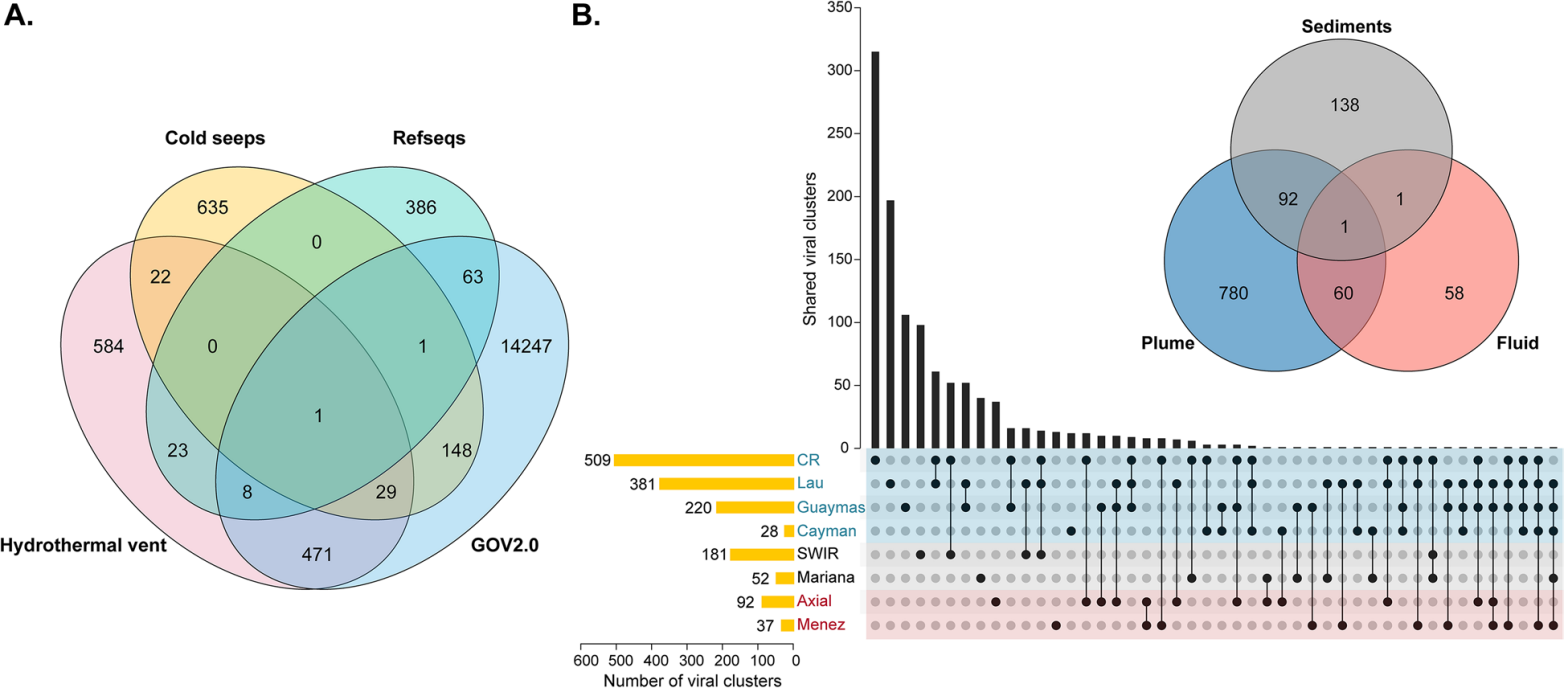
Distribution of viral clusters determined by gene-sharing network analysis. **A** Venn diagram of shared viral clusters among different environmental virus datasets and RefSeq. **B** UpSet plots of shared viral clusters among different hydrothermal vent sites

To gain further insight into this viral diversity and distribution, we then used an extensively validated, network-based method [56] to investigate the relationship among hydrothermal vent vOTUs and viral sequences identified from other marine ecosystems. The 4662 vOTUs recovered from hydrothermal vents were compared to NCBI Viral RefSeq v97 and viral contigs in other marine metagenomic datasets: GOV 2.0 seawater [57] and cold seeps [58]. vConTACT2 was used to de novo predict genus-level groups (viral clusters, VCs) from viral population data [56]. Lastly, a total of 16,618 clusters were generated, reflecting the huge and unexplored diversity of marine viruses (Fig. 5A). As the largest marine virus database to date, the GOV 2.0 datasets contributed the largest number of VCs (14,968 clusters), while taxonomically known viruses from NCBI RefSeq only formed 482 clusters. Consistent with previous studies [58], the viral compositions in different habitats varied considerably, with only 30 clusters shared by all three marine metagenomic datasets (Fig. 5A).

The 4662 hydrothermal vent vOTUs were grouped into 1138 genus-level clusters, of which only 32 VCs contained genomes of known viruses from NCBI RefSeq. Moreover, 584 VCs (~51%) were exclusively composed of hydrothermal vent viruses, which may represent completely new genus candidates (Fig. 5A). These VCs contained 1107 vOTUs, most of which were ssDNA viruses within Microviridae (548 vOTUs) and the CRESS-DNA virus families (122). Approximately one-third of the vOTUs (314) could be classified as tailed phages, probably of novel genera within *Myoviridae* (144), Podoviridae (65), Siphoviridae (57), and unclassified *Caudovirales* (48). The remaining 123 vOTUs could not be taxonomically classified at the family or even higher levels.

Within hydrothermal vent habitats, a high proportion of the viruses seemed to be endemic, given that a majority (818 VCs, 71.9%) of the VCs only occurred in a specific vent site (Fig. 5B). This observation was consistent with previous work showing that most viruses in hydrothermal vent fluids have limited distributions [31]. Generally, the VC richness in hydrothermal plumes was higher than those in sediments or diffuse fluids, suggesting a greater viral diversity in hydrothermal plumes. Although the viral communities of different hydrothermal plume samples were somewhat similar at the family level, they shared a small fraction of clusters. The number of clusters shared between sediments and fluid was even lower, and only one cluster was detected across all sample types (Fig. 5B). The cluster turned out to be viruses in the family Microviridae, reinforcing the ubiquity of this group.

### Virus-host connections in hydrothermal vents

**Fig. 6:**
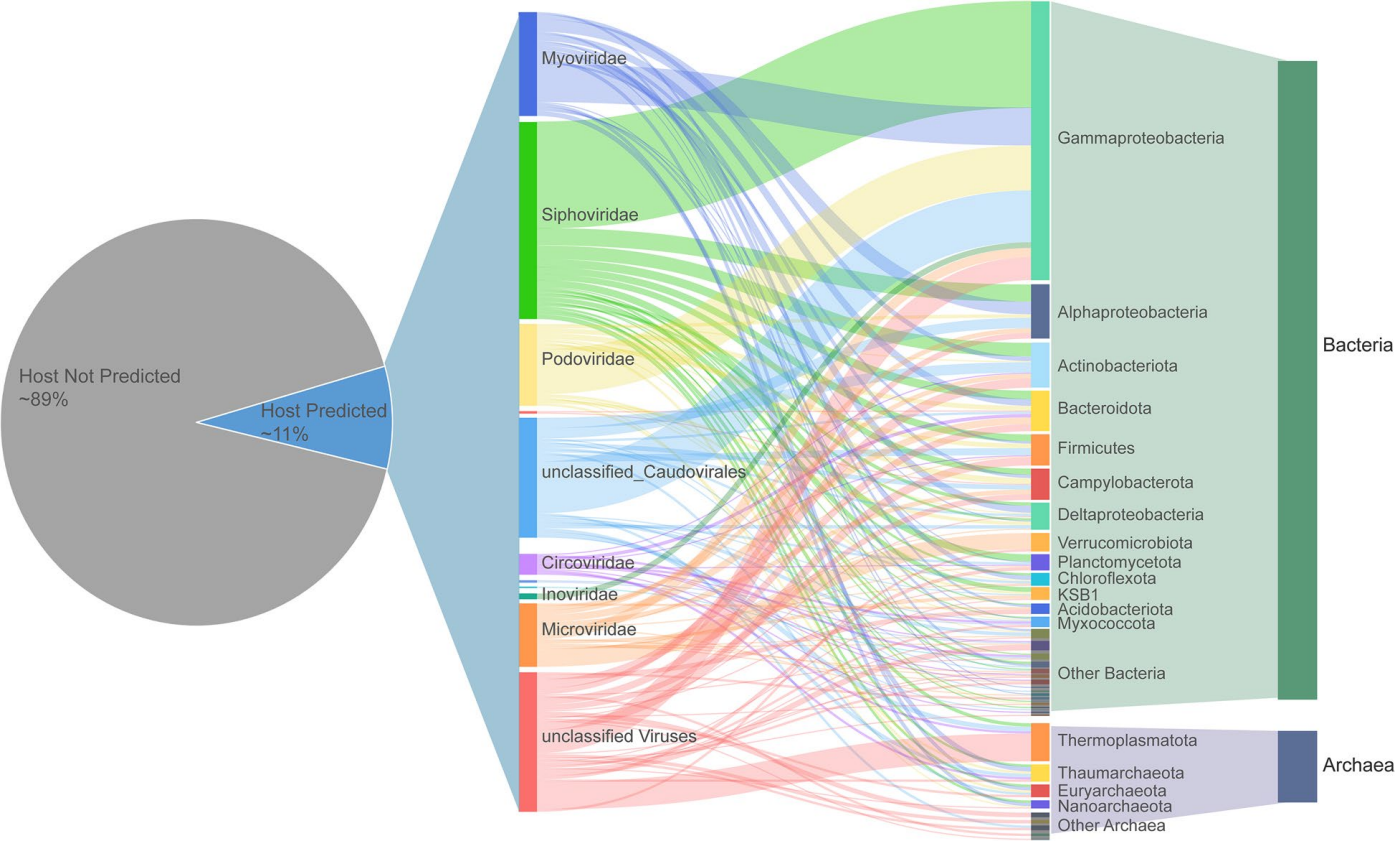
Predicted virus-host linkages in hydrothermal vents

The interactions between viruses and their hosts exert a strong influence on microbial diversity and are essential for understanding the ecology and functioning of microbial communities [16]. We sought to link the hydrothermal vent vOTUs to their potential hosts by using a combination of four in silico methods based on their CRISPR spacer match, tRNA match, sequence similarity, and k-mer frequencies [35]. As a result, putative targeted hosts were predicted for a small fraction (494 vOTUs, ~11%) of the hydrothermal vent vOTUs (Fig. 6, Supplementary Table 3). Specifically, 230 vOTUs were linked to MAGs (Supplementary Fig. 1B) and SAGs derived from hydrothermal vents. Most connections were predicted by WIsH and CRISPR spacer matching (252 viral-host pairs each), 114 pairs by sequence homology, and 119 pairs by tRNA matching. Among them, the linkages of 78 viral-host pairs were supported by two or more prediction strategies. The majority (~89%) of these vOTUs were predicted to infect a specific host, and only 31 OTUs were linked to hosts from different prokaryotic phyla. This result is consistent with the common perceptions and previous findings that most viruses have a narrow host range [35, 40, 58].

The predicted hosts of hydrothermal vent phages include bacterial and archaeal species from 39 different phyla (Fig. 6, Supplementary Table 3). Seventy-nine vOTUs were linked to archaea, of which the phylum *Candidatus* Thermoplasmatota was the most frequently predicted (33 associated vOTUs). This newly proposed phylum contains the Marine group II (MGII) and Marine group III (MGIII) archaea [59]. MGII dominate ocean surface waters and may play important roles in the marine carbon cycle [60], whereas members of MGIII live in deep mesopelagic and bathypelagic environments at relatively low abundance [61]. Both of these groups have been found in deep-sea hydrothermal vents and are thought to contribute to organic compound degradation [62].

*Gammaproteobacteria* were the most frequently predicted bacterial hosts, with 144 associated vOTUs, followed by *Actinobacteria* (39 vOTUs), *Alphaproteobacteria* (37 vOTUs), Bacteroidota (34 vOTUs), *Firmicutes* (27 vOTUs), and *Campylobacterota* (26 vOTUs). These groups were among the most abundant and active bacterial lineages in the hydrothermal vent ecosystems, as previously reported [9, 12] and as revealed in this study (Supplementary Fig. 1A). For example, *Gammaproteobacteria* is a large bacterial class with metabolic versatility and is observed in almost all habitats surrounding hydrothermal vents [63]. We found that the most frequently predicted hosts within *Gammaproteobacteria* were the genera *Acinetobacter* (with 15 associated vOTUs), *Alteromonas* (15 vOTUs), *Pseudomonas* (14 vOTUs), and *Alcanivorax* (8 vOTUs), which were dominant in most of the samples involved in this study. According to the well-known kill-the-winner hypothesis [16], abundant microbes are more likely to be infected and lysed by viruses because a high population density will increase the host-virus encounter rate [64, 65]. Thus, it is not surprising that many viruses target *Gammaproteobacteria* in hydrothermal vents. These viruses showed high abundances (Supplementary Fig. 4) and might play important roles in regulating the vent microbial communities.

As another abundant and ubiquitous group that inhabits hydrothermal vent environments, *Campylobacterota* is a group of chemolithotrophic primary producers that primarily use sulfur compounds and hydrogen as electron donors [66] and are regarded as indicators of hydrothermal activity and passive tracers of vent fluids [9, 63, 67]. However, only a few potential prophage regions have been reported in the complete genomes of deep-sea *Campylobacterota* [68, 69], and only one of them has been isolated [70] to date. In this study, 26 vOTUs were shown to be potentially able to infect members of the phylum *Campylobacterota*, particularly the genus *Sulfurimonas* (14 associated vOTUs). These findings included viruses from the families Myoviridae, Podoviridae, Siphoviridae, Herelleviridae, and Microviridae, while 8 vOTUs remained unclassified at the family level, indicating the unrevealed diversity of viruses infecting hydrothermal vent Campylobacterota. Further analysis of these viral genomes will provide new insight into the interactions of this ecologically important group and their phages.

CRESS-DNA viruses from the existing families were reported to infect hosts across the eukaryotic domain, including plants, fungi, and animals [49], but a recent study based on CRISPR analysis suggested that viruses from the CRESS-DNA family Smacoviridae infected methanogenic archaea instead of humans [71]. Interestingly, our results also revealed some potential connections between CRESS-DNA viruses and bacterial or archaeal hosts (Fig. 6, Supplementary Table 3), suggesting a broader host range for this group. To date, CRESS-DNA virus isolates with definitive hosts include members from five CRESS-DNA families [71]. However, the number of CRESS-DNA viruses discovered in metagenomics surveys now far exceeds the number of biologically characterized viral isolates [49]. Considering the diversity of CRESS-DNA viruses [72, 73] and their origin from bacterial rolling circle-replicating plasmids [74, 75], it is possible that some CRESS-DNA viruses infect hosts beyond eukaryotes.

### AMGs of vent viruses are involved in various metabolic pathways

**Fig. 7:**
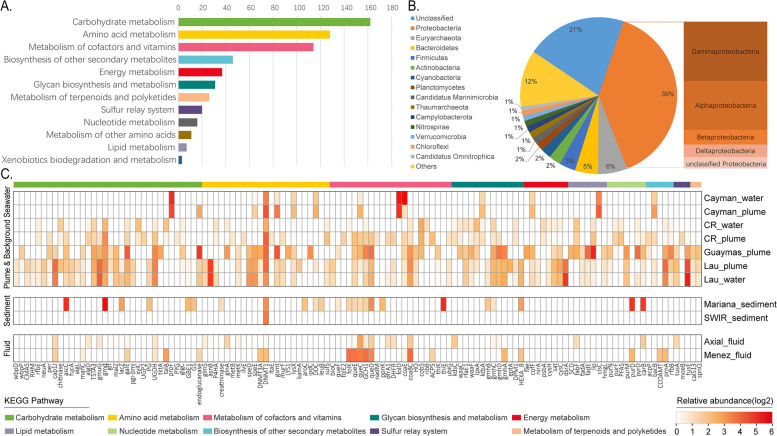
Function and abundance profiles of virus-encoded auxiliary metabolic genes (AMGs). **A** Classification of AMGs into KEGG metabolic categories. **B** Predicted source organisms of viral AMGs. **C** Relative abundance of AMGs in different hydrothermal vent samples

Viral infections can affect host metabolism via the expression of viral AMGs. To better understand the ecological impact of viruses in deep-sea hydrothermal ecosystems, we searched the AMGs in hydrothermal vent viral genomes and calculated their relative abundances. Based on the comprehensive annotation of viral ORFs, a total of 608 genes were considered to be putative AMGs (Fig. 7, Supplementary Table 4). Sequence homology searches against the NCBI NR database showed that a large proportion of these AMGs were probably acquired from *Proteobacteria*, especially the *Gammaproteobacteria* and *Alphaproteobacteria*, while ~21% of the AMGs came from unclassified source species (Fig. 7B). The origins of AMGs reflect virus-host connections, because phages generally acquire AMGs from their hosts [76]. According to the KEGG annotation, the identified AMGs of hydrothermal vent viruses were involved in a variety of metabolic pathways, including those related to carbohydrate metabolism, amino acid metabolism, and the metabolism of cofactors and vitamins (Fig. 7A). This trend is consistent with the viral metabolic profiles revealed by analyzing 6 hydrothermal vent metagenomes [41], suggesting that there are some common features in the patterns of metabolic capabilities of hydrothermal vent viromes.

The AMG composition and abundance profiles across the hydrothermal vent samples indicated that viruses in hydrothermal plumes encoded a larger number of AMGs with diverse functions (Fig. 7C). Most of the AMGs had higher abundances in the plume samples compared with hydrothermal fluid or sediments. This observation may reflect the role of viruses in facilitating host adaptation to the dynamic nature of hydrothermal plumes and is in congruence with the metabolic versatility of microbes therein [10]. Specifically, AMGs involved in energy metabolism were found only in plume samples, including those related to methane, nitrogen, and sulfur metabolism (Fig. 7C). Several AMGs were associated with sulfur metabolism pathways, such as genes coding for phosphoadenosine phosphosulfate reductase (*cysH*), adenylylsulfate kinase (*cysC*), sulfate adenylyltransferase (*sat*), and dissimilatory sulfite reductase subunits A (*dsrA* or *rdsrA*, as referred to in a previous study [25]). The *cysH* and *cysC* genes are involved in assimilatory sulfate reduction, whereas the *sat* and *dsrA* genes are related to dissimilatory sulfur reduction/oxidation [77]. Sulfur oxidation and sulfate reduction are both important parts of sulfur cycling in hydrothermal vent ecosystems [78], and the presence of these AMGs suggested that phages participate extensively in these pathways.

The most abundant AMG identified in hydrothermal vent metagenomes was DNA cytosine methyltransferase (DNMT1, *dcm*). However, viral *dcm* and another 13 AMGs were present in metagenomes derived from several diverse environments and were thus considered to perform central functions in the viral life cycle [38]. Except for these globally conserved AMGs, the thiouridine synthase subunit E (*tusE*, a homolog of *dsrC*) gene related to the sulfur relay system was identified in high abundance. This gene encodes a sulfur transfer protein for tRNA thiol modifications, which is required for protein synthesis machinery [79]. The expression of tRNA thiolation genes has been reported to increase the stability of tRNA structure and is essential for bacterial survival at high temperatures [80, 81]. In addition, it has been suggested that the sulfur relay system is involved in microbial tolerance against acid stress [82, 83], heavy metals [84], and organic solvents [85]. Therefore, this AMG may benefit the hosts by improving their adaptability to various stress conditions and thus provide them with a growth advantage in hydrothermal vent environments in which the temperature and chemistry fluctuate substantially.

It is worth noting that some of the predicted AMGs may not be *bona fide* AMGs. First, it is difficult to avoid false-positive predictions of viral contigs completely since these metagenomes were derived from cellular fractions. Although the candidate AMGs were co-localized with at least one viral hallmark gene, they might belong to the host regions that were retained due to the miscall of a prophage boundary [32]. Another concern is the true function of candidate AMGs. As described above, some metabolic genes are more likely to be involved in the viral life cycle rather than the host metabolism, such as methyltransferases, glycosyl transferases, glycoside hydrolases, and adenylyltransferases [86–88]. Thus, further investigations, such as genome context assessments and functional analyses of putative AMGs, are required for a better understanding of the viral impacts on hydrothermal vents.

## Conclusions

In this study, we explored the viral community of CR hydrothermal vents and additional hydrothermal vent sites across a wide geographical area based on metagenomic data. We found that deep-sea hydrothermal systems are large reservoirs of novel viruses. Both vent prokaryotic and eukaryotic communities are affected by viruses that target diverse hosts and modulate their metabolisms. These interactions shape the structure and function of microbiomes in deep-sea hydrothermal vents and may profoundly influence the broader oceans via vent fluid circulation and plume drift. Our exploration highlights global vent viral diversity and suggests the significant roles of viruses in this unique ecosystem. With improvements in deep-sea sampling and culture-dependent and culture-independent technologies, a more comprehensive understanding of virus-host interactions in hydrothermal ecosystems is soon to be expected.

## Methods

### Sample collection

Plume and background seawater samples were collected from two different deep-sea hydrothermal vents, “Wocan” and “Tianxiu,” at the Carlsberg Ridge of the northwest Indian Ocean during the COMRA cruise DY 38 in March 2017 (Supplementary Table 1). The human-operated vehicle “Jiaolong” was used to collect 1.5-L water samples in individual Niskin bottles. The collected water was filtered through 0.22 μm polycarbonate membranes (diameter 45 mm; Whatman, Clifton, NJ, USA) and frozen at −80 °C on board for DNA extraction. For the single-cell sequencing, plume samples from the “Wocan” vent were fixed with glycerol-Tris-EDTA buffer [89] and frozen at −80 °C until further processing.

### DNA extraction and metagenomic sequencing

The total DNA was extracted from filtration membranes as described previously [7]. Multiple displacement amplification of genomic DNA was performed using the illustra Ready-To-Go GenomiPhi V3 DNA Amplification Kit (GE Healthcare, Piscataway, NJ, USA). Paired-end library was constructed using NEXTFLEX Rapid DNA-Seq (Bioo Scientific, Austin, TX, USA). Adapters containing the full complement of sequencing primer hybridization sites were ligated to the blunt end of fragments. Shotgun sequencing was performed on Illumina HiSeq PE150 platform (Illumina Inc., San Diego, CA, USA) at Majorbio Bio-Pharm Technology Co., Ltd. (Shanghai, China) according to the manufacturer’s instructions (www.illumina.com).

### Metagenome assembly and annotation

The raw reads obtained by Illumina paired-end sequencing were trimmed and quality filtered using fastp software [90]. Clean reads were then assembled using MEGAHIT [91] with default options. Metagene [92] was used to predict protein coding sequences (CDS) from the metagenomic assemblies. Nonredundant genes generated by CD-HIT clustering (with 95% shared sequence identity and 90% coverage) were aligned to the NCBI NR database using BLASTp (with an *e*-value cut-off of 1e-5) for taxonomic classification. Functional annotations were conducted based on comparisons with KEGG [93], eggNOG v5.0 [94], and the Carbohydrate-Active enZYmes (CAZy) databases [95] using the BLASTp [96] program (with an *e*-value cut-off of 1e-5). Additionally, 29 publicly available metagenomic datasets generated from hydrothermal vent samples (Supplementary Table 1) were downloaded from the NCBI Sequence Read Archive (SRA) database and were quality controlled, assembled, and annotated as described above.

### Metagenomic binning and metagenome-assembled genome (MAG) classification

Contigs larger than 1500 bp from the final assemblies were included for metagenomic binning using MetaBAT2 v2.12.1 [97] with default parameters. The original bins were then run through MetaWRAP’s reassemble_bins module [98] to improve their quality, and the completeness and contamination of the resulting bins were evaluated by CheckM [99]. The high- and medium-quality bins (completeness ≥ 50% and contamination ≤ 10%) were then dereplicated at 95% average nucleotide identity (ANI) using dRep v2.3.2 [100], resulting in 581 species-level MAGs. The taxonomic assignment of the MAGs was performed using the GTDB-Tk package v0.3.2 [101] and was ultimately converted into the corresponding NCBI taxonomy. The phylogenomic relationships of MAGs were inferred using IQ-TREE 2 [102] based on a concatenation of 120 bacterial or 122 archaeal marker genes identified by GTDB-Tk. Support for nodes in the ML trees was evaluated with 1000 ultrafast bootstrap replicates [103], and the generated tree was visualized using iTOL v4 [104].

### SAG library construction, sequencing, and analysis

The sequencing of plume-derived SAGs was performed in the Bigelow Laboratory Single Cell Genomics Center (https://scgc.bigelow.org/). Fluorescence-activated cell sorting, cell lysis, multiple displacement amplification, Illumina sequencing, and de novo genome assembly were conducted as previously described [89]. The quality assessment and taxonomic classification of SAGs were performed as described above for MAGs.

### Identification of viral contigs

Contigs ≥ 2 kb from metagenome assemblies were used to recover viral sequences. VirSorter analysis [40] was run with the parameter “--db 2 (viromes database),” and only the highest confidence contig categories 1, 2, 4, and 5 were included in this study, with categories 4 and 5 being manually curated. Contigs containing at least one of the viral hallmark genes (such as “virion structure,” “capsid,” “portal,” “head,” “tail,” “baseplate,” or “terminase”) were retained. VIBRANT v1.2.1 [41] was also used to identify viral contigs using the default parameters, and only the complete circular, high- and medium-quality drafts were kept for further analysis. The contigs identified by VirSorter and VIBRANT were then compiled and clustered at 95% shared nucleotide identity and 80% coverage [105], yielding 4662 viral populations, or viral operational taxonomic unit (vOTUs). Lastly, the CheckV pipeline was used to estimate the completeness of the viral genomes and to predict viral lifestyles [44].

### Abundance profiling in metagenomics data

For taxonomic profiling of prokaryotic communities, 16S miTags were recovered from the metagenomic reads using phyloFlash [36]. The extracted 16S miTags were mapped against the SILVA SSU Ref. database (v132) [106] for taxonomic assignment. To calculate the relative abundances of vOTUs and host microorganisms in each sample, clean reads from metagenomes were mapped to the viral contigs or microbial genomes using the CoverM package (https://github.com/wwood/CoverM) with contig mode and genome mode, respectively. RPKM (reads per kilobase per million mapped reads) values were selected to represent the relative abundances of the viral and host populations. Pearson correlation was used to calculate the distances between samples for hierarchical clustering. Heatmaps were generated using the pheatmap R package and TBtools [107].

### Host prediction

Four computational host prediction strategies were used to identify virus-host interactions [35]. (i) CRISPR spacers match: a clustered regularly interspaced short palindromic repeats (CRISPRs) spacer database was created for a set of microbial genomes using the MinCED tool [108]. For metagenomics reads, Crass v1.0.1 [109] was used with the default parameters to recover CRISPR spacers and repeat elements. The identified spacers were queried for exact sequence matches against all viral contigs using the BLASTn-short mode in the BLAST+ package [110]. Match requirements were at least 95% identity over 95% spacer length, and only ≤ 1 mismatch was allowed. The corresponding CRISPR direct repeat types were connected to microbial genomes via BLASTn (with an *e*-value cut-off of 1e-10, 100% nucleotide identity) [111]. (ii) Transfer RNA (tRNA) match: tRNAs were recovered from the microbial genomes and viral contigs using ARAGORN with the “−t” option [112]. The identified tRNA sequences were compared using BLASTn, and only a perfect match (100% coverage and 100% identity) was considered indicative of putative host-virus pairs. (iii) Nucleotide sequence homology search [113]: To link prophages with hosts, viral contigs were searched against microbial genomes using BLASTn with the following thresholds: 75% minimum coverage of the viral contig length, 70% minimum nucleotide identity, 50 minimum bit score, and 0.001 maximum *e*-value. (iv) k-mer frequencies: WIsH v1.0 [114] was run with the default parameters against the host database. Connections were inferred when *p* < 0.001. If multiple hosts were predicted for a vOTU, the one supported by different approaches was chosen as the one with the most confidence. The host database employed for these prediction methods was composed of (i) all reference genomes from the Genome Taxonomy Database (GTDB), (ii) all MAGs (≥ 50% completeness, and ≤ 10% contamination) recovered from hydrothermal vent metagenomes (*n* = 581), (iii) all SAGs obtained from the CR hydrothermal vent (*n* = 440), and (iv) a custom collection of marine microbial genomes from the Marine Culture Collection of China (*n* = 1452).

### Viral taxonomic assignment and network analysis

The predicted open reading frames (ORFs) of the viral contigs were mapped against the NR protein database using DIAMOND v0.9.21 [115], and their taxonomic affiliations were determined using the CAT v5.0.3 package [116] based on the last common ancestor (LCA) algorithm. Contig classification is based on a voting approach of all classified ORFs by summing up all the bit scores from ORFs supporting a specific classification. Protein-sharing network analysis of the hydrothermal vent vOTUs, the reference phage genomes (from NCBI Viral RefSeq version 97), the Global Oceans Viromes 2 (GOV 2.0) datasets [57], and viral contigs from cold seeps [58] was performed using vConTACT v2.0 [56]. Briefly, Prodigal v2.6.3 [117] was used for ORF prediction from the vOTUs. The predicted protein sequences were then subjected to all-to-all BLASTp using DIAMOND, and the BLAST result file was used as input for vConTACT2. The similarity score between vOTUs was calculated based on the number of shared protein clusters, and related vOTUs with a similarity score of ≥ 1 were grouped into viral clusters.

### Construction of phylogenetic trees

For phylogenetic trees, the deduced amino acid sequences of selected marker genes were aligned using the MUSCLE program [118], and the multiple alignments were trimmed with TrimAl v1.2 [119]. IQ-TREE2 [102] was used to infer the maximum-likelihood (ML) tree with the best substitution model selected by ModelFinder [120], and support for nodes in the ML trees was evaluated with 1000 ultrafast bootstrap replicates [103]. The resulting trees were visualized and annotated using FigTree v1.4.4 (http://tree.bio.ed.ac.uk/software/figtree/) and the iTOL v4 online tool [104].

### Identification of auxiliary metabolic genes

Functional annotations on the ORFs in the viral contigs were conducted based on comparisons with the eggNOG v5.0 [94] database using eggNOG-mapper v2 [121]. Genes with a KEGG annotation falling under the “metabolic pathways” category or “sulfur relay system” were considered to be putative vAMGs, as previously defined in VIBRANT [41]. Lastly, manual curation was performed to remove the metabolic genes involved in common viral functions. To generate the abundance profiles for AMGs, clean reads were mapped to the metagenomes using bowtie2 [122], and the RPKM values of each gene were calculated. The sum of the RPKM values of genes with the same KO annotations was used to represent the relative abundance of each gene category.

## Supplementary Information

**Additional file 1: Supplementary Table 1.** Metagenome datasets used for analysis in this study.

**Additional file 2: Supplementary Figure 1.** Microbial communities in deep-sea hydrothermal vents. (A) Relative abundance of 16S miTag in 34 hydrothermal vent samples. The top 30 most abundant phyla (class level for Proteobacteria) among the metagenomes are shown. (B) Phylogenetic tree of high-quality metagenome-assembled genomes (MAGs) recovered from 34 hydrothermal vent metagenomes. Maximum-likelihood phylogenetic trees of bacterial and archaeal MAGs at the phylum level (class level for Proteobacteria) were inferred from 120 bacterial or 122 archaeal single-copy marker genes, respectively. Support for nodes in the ML trees was evaluated with 1000 ultrafast bootstrap replicates, and bootstrap scores >70% are flagged with dots. The number of MAGs related to viruses and the total number of recovered MAGs in the clade are shown in brackets.

**Additional file 3: Supplementary Table 2.** Characteristics of vOTUs recovered from hydrothermal vent metagenomes. The taxonomic affiliation of vOTUs was inferred using the last common ancestor algorithm in CAT v5.0.3. The completeness of the viral genomes and viral lifestyles were determined using the CheckV pipeline.

**Additional file 4: Supplementary Figure 2.** Genome quality and taxonomic composition of hydrothermal vent vOTUs. (A) Proportion of genome quality categories assessed by CheckV. (B) Taxonomic classification of vOTUs at the family level.

**Additional file 5: Supplementary Figure 3.** Distribution patterns of all hydrothermal vent vOTUs. The relative abundances of vOTUs (y-axis) in each sample (x-axis) were calculated as reads per kilobase per million mapped reads (RPKM values) and were normalized on the log2 scale. The vOTUs and the samples were hierarchically clustered.

**Additional file 6: Supplementary Table 3.** Predicted virus-host connections for vOTUs. The highest confidence host for each vOTU is highlighted.

**Additional file 7: Supplementary Figure 4.** Distribution patterns of viruses and their predicted hosts in deep-sea hydrothermal vents. The relative abundances of vOTUs (top left triangle) and their predicted hosts (bottom right triangle) were grouped by host taxonomy and were normalized on the log2 scale.

**Additional file 8: Supplementary Table 4.** Annotation of putative auxiliary metabolic genes.

## Data Availability

The metagenomic datasets used for analysis in this study are publicly available in the NCBI SRA repository at https://www.ncbi.nlm.nih.gov/sra, and accession numbers are listed in Supplementary Table 1. Metagenomic data from CR hydrothermal vent were also deposited in the SRA database (accession number PRJNA685608). The sequences of the vOTUs generated from the current study have been deposited in the National Omics Data Encyclopedia (NODE) database at https://www.biosino.org/, accession number OEP003015.
