# Supplementary figures and images for: Virus diversity and interactions with hosts in deep-sea hydrothermal vents
Source: Microbiome. 2022 Dec 24;10:235. doi: 10.1186/s40168-022-01441-6 (PMC9789665; doi:10.1186/s40168-022-01441-6)

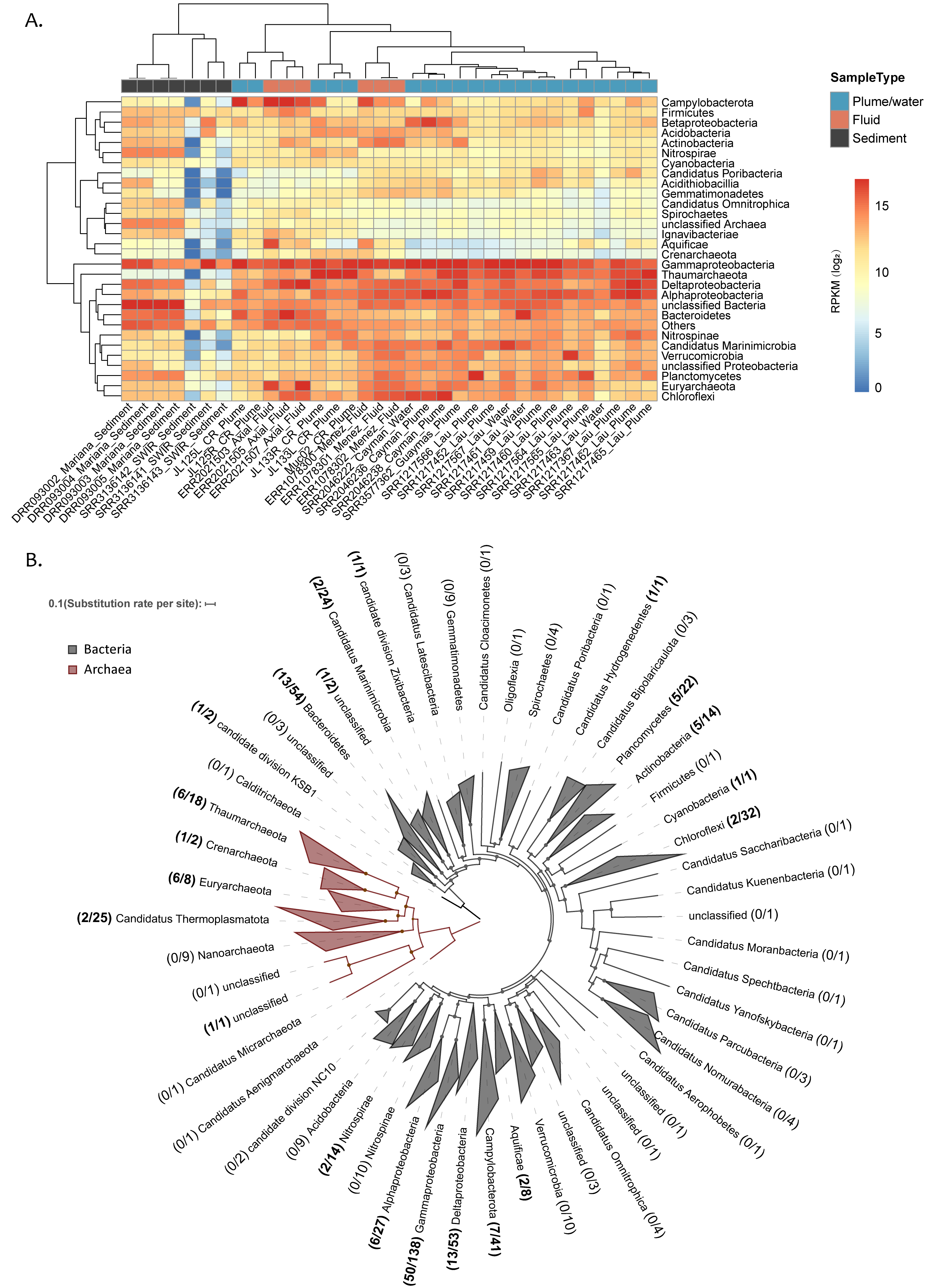

Supplement: Supplementary file 3 — Additional file 2: Supplementary Figure 1. Microbial communities in deep-sea hydrothermal vents. (A) Relative abundance of 16S miTag in 34 hydrothermal vent samples. The top 30 most abundant phyla (class level for Proteobacteria) among the metagenomes are shown. (B) Phylogenetic tree of high-quality metagenome-assembled genomes (MAGs) recovered from 34 hydrothermal vent metagenomes. Maximum-likelihood phylogenetic trees of bacterial and archaeal MAGs at the phylum level (class level for Proteobacteria) were inferred from 120 bacterial or 122 archaeal single-copy marker genes, respectively. Support for nodes in the ML trees was evaluated with 1000 ultrafast bootstrap replicates, and bootstrap scores >70% are flagged with dots. The number of MAGs related to viruses and the total number of recovered MAGs in the clade are shown in brackets. [file 40168_2022_1441_MOESM2_ESM.tif]

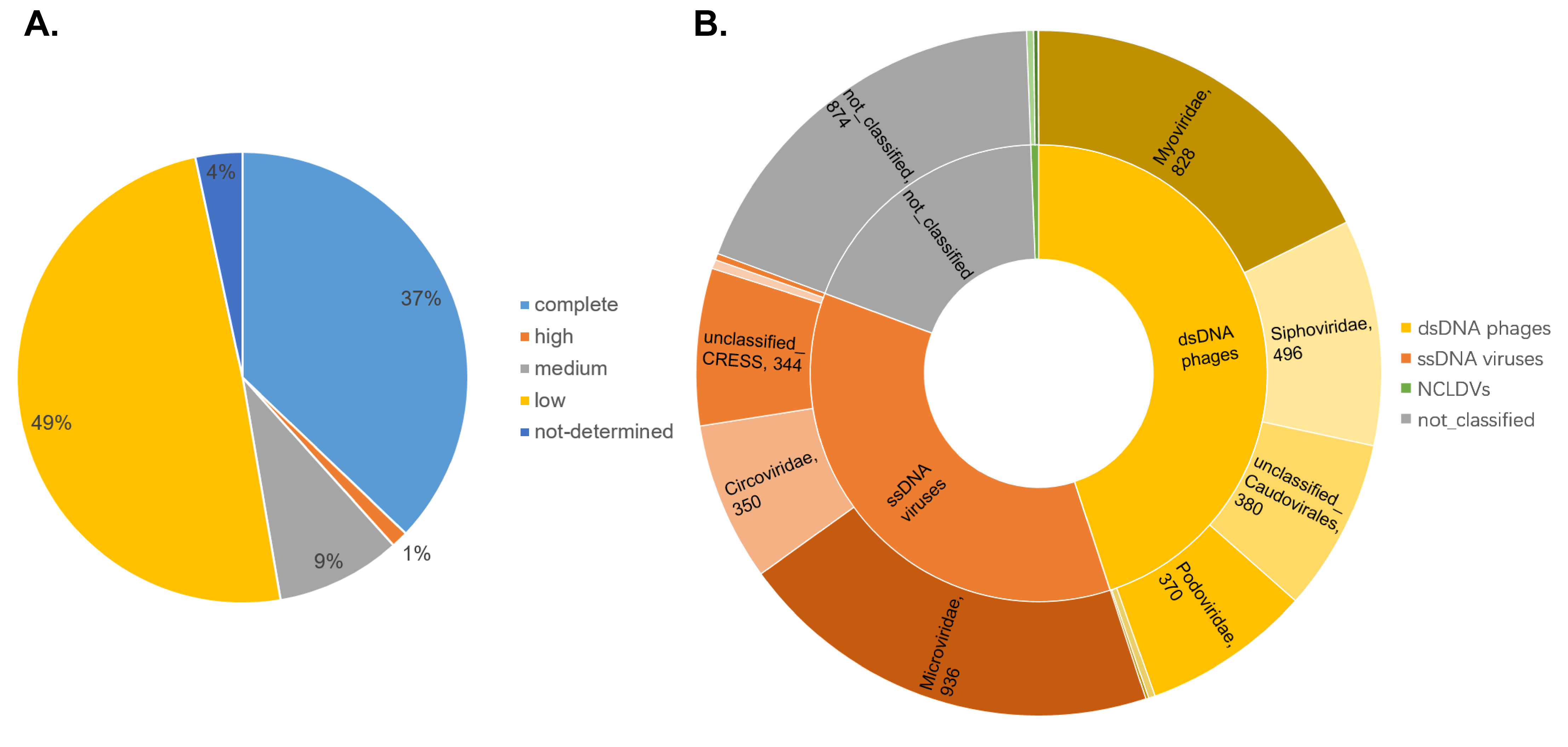

Supplement: Supplementary file 5 — Additional file 4: Supplementary Figure 2. Genome quality and taxonomic composition of hydrothermal vent vOTUs. (A) Proportion of genome quality categories assessed by CheckV. (B) Taxonomic classification of vOTUs at the family level. [file 40168_2022_1441_MOESM4_ESM.tif]

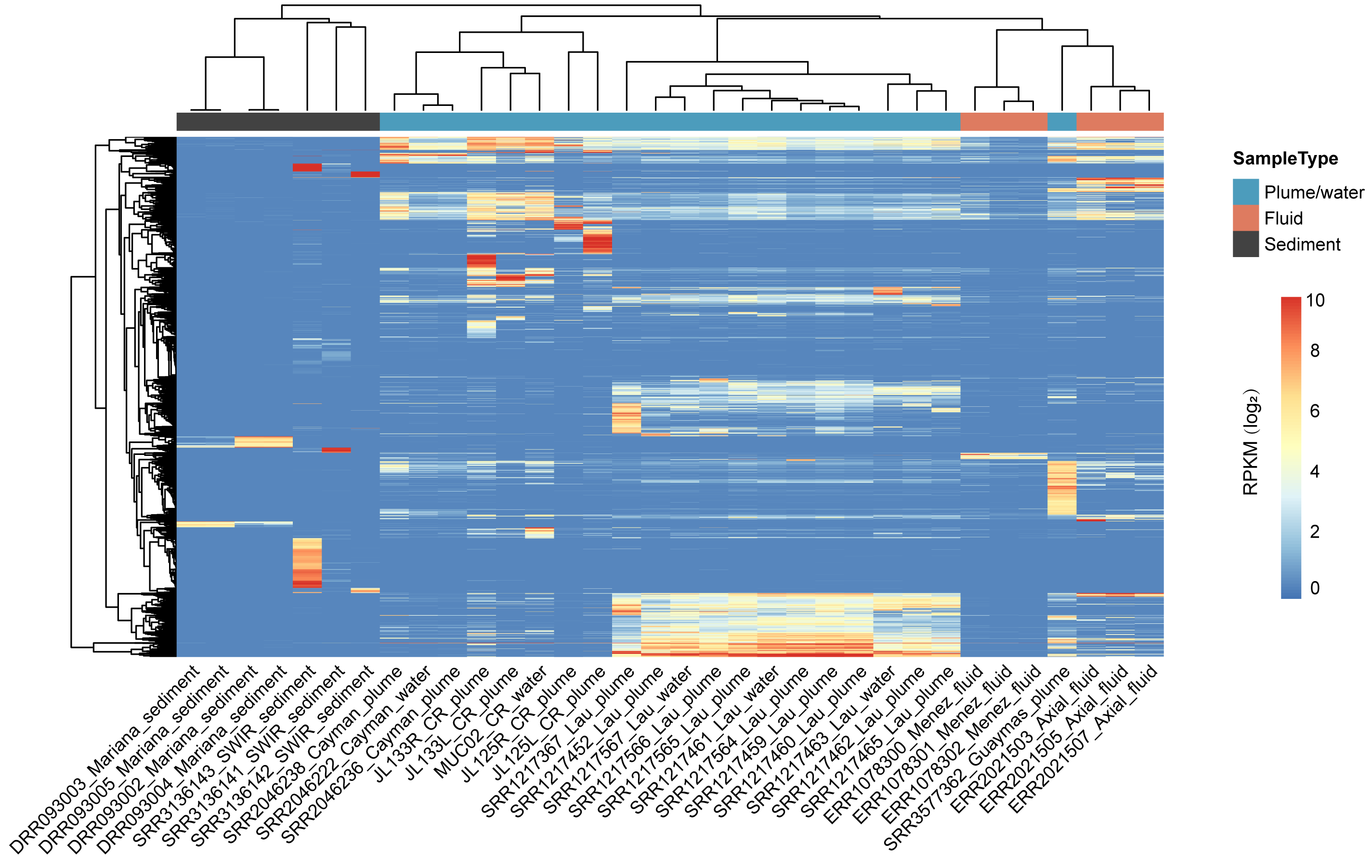

Supplement: Supplementary file 6 — Additional file 5: Supplementary Figure 3. Distribution patterns of all hydrothermal vent vOTUs. The relative abundances of vOTUs (y-axis) in each sample (x-axis) were calculated as reads per kilobase per million mapped reads (RPKM values) and were normalized on the log2 scale. The vOTUs and the samples were hierarchically clustered. [file 40168_2022_1441_MOESM5_ESM.tif]

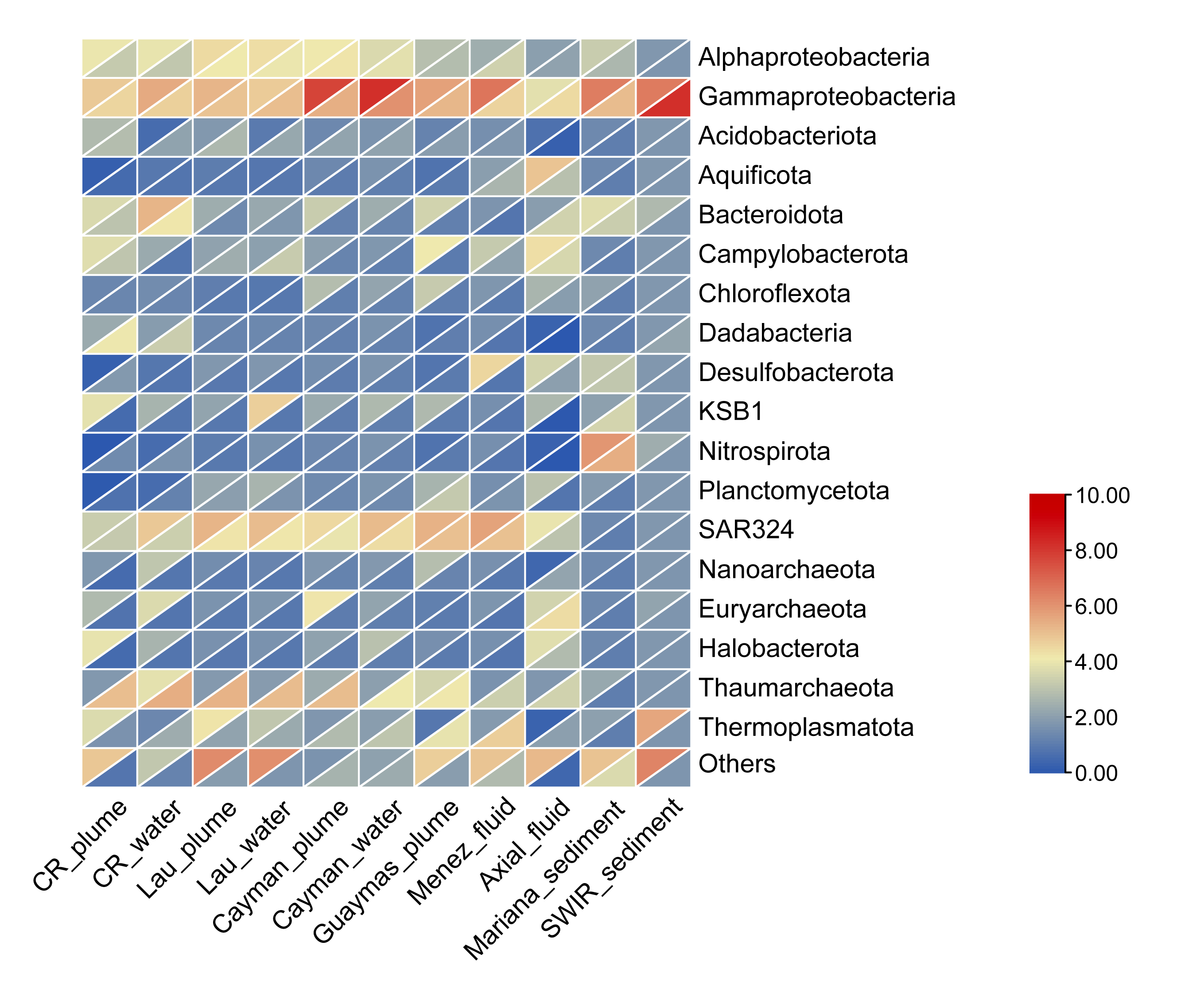

Supplement: Supplementary file 8 — Additional file 7: Supplementary Figure 4. Distribution patterns of viruses and their predicted hosts in deep-sea hydrothermal vents. The relative abundances of vOTUs (top left triangle) and their predicted hosts (bottom right triangle) were grouped by host taxonomy and were normalized on the log2 scale. [file 40168_2022_1441_MOESM7_ESM.tif]
